# Levels of pro-apoptotic regulator Bad and anti-apoptotic regulator Bcl-xL determine the type of the apoptotic logic gate

**DOI:** 10.1186/1752-0509-7-67

**Published:** 2013-07-24

**Authors:** Marta N Bogdał, Beata Hat, Marek Kochańczyk, Tomasz Lipniacki

**Affiliations:** 1 Institute of Fundamental Technological Research, Polish Academy of Sciences, Warsaw 02-106, Poland; 2 Department of Mathematics, Informatics and Mechanics, University of Warsaw, Warsaw 02-097, Poland; 3 Department of Statistics, Rice University, Houston, TX 77025, USA

**Keywords:** Apoptosis, Cell survival, Signaling pathway, Bcl-2 family, Bistability, Boolean logic, Ordinary differential equations

## Abstract

**Background:**

Apoptosis is a tightly regulated process: cellular survive-or-die decisions cannot be accidental and must be unambiguous. Since the suicide program may be initiated in response to numerous stress stimuli, signals transmitted through a number of checkpoints have to be eventually integrated.

**Results:**

In order to analyze possible mechanisms of the integration of multiple pro-apoptotic signals, we constructed a simple model of the Bcl-2 family regulatory module. The module collects upstream signals and processes them into life-or-death decisions by employing interactions between proteins from three subgroups of the Bcl-2 family: pro-apoptotic multidomain effectors, pro-survival multidomain restrainers, and pro-apoptotic single domain BH3-only proteins. Although the model is based on ordinary differential equations (ODEs), it demonstrates that the Bcl-2 family module behaves akin to a Boolean logic gate of the type dependent on levels of BH3-only proteins (represented by Bad) and restrainers (represented by Bcl-xL). A low level of pro-apoptotic Bad or a high level of pro-survival Bcl-xL implies gate AND, which allows for the initiation of apoptosis only when two stress stimuli are simultaneously present: the rise of the p53 killer level and dephosphorylation of kinase Akt. In turn, a high level of Bad or a low level of Bcl-xL implies gate OR, for which any of these stimuli suffices for apoptosis.

**Conclusions:**

Our study sheds light on possible signal integration mechanisms in cells, and spans a bridge between modeling approaches based on ODEs and on Boolean logic. In the proposed scheme, logic gates switching results from the change of relative abundances of interacting proteins in response to signals and involves system bistability. Consequently, the regulatory system may process two *analogous* inputs into a *digital* survive-or-die decision.

## Background

### Biological background

Apoptosis is a programmed cell death required for removal of infected, damaged or unwanted cells [1]. It assists in the development and aging as a homeostatic mechanism controlling cell populations in tissues, and it constitutes a key immune defense mechanism against infected or cancerous cells [1,2]. Disrupted regulation of apoptosis contributes to carcinogenesis, spread of infection, autoimmunological and neurodegenerative disorders [3-5]. Since there could be many reasons for which a cell should be eliminated, there exist numerous pathways (major of them discussed below) through which apoptosis can be initiated. This opens an interesting question how the pro-apoptotic, but also pro-survival, signals converge and are integrated before the survive-or-die decision is reached.

In mammalian cells, apoptosis can be induced via two classes of apoptotic pathways: extrinsic and intrinsic [6,7]. In both classes, signaling leads to the activation of a family of cysteine proteases named caspases which serve as executors of the apoptotic process [8,9]. Caspases are present in virtually every cell in the form of inactive precursors called pro-caspases [9]. Each apoptotic pathway activates some initiator caspases which, in turn, activate the main executioner, caspase-3 [8]. The release of cytochrome *c* from mitochondria results in the formation of apoptosome (containing also Apaf-1 and caspase-9) and activation of caspase-9 [10], which activates caspase-3 triggering the caspase cascade. This cascade involves caspase-2, -6, -8 and -10 [11,12] responsible for the proteolytic dismantling of the apoptotic cell [13]. Caspase-3 is responsible for the further release of cytochrome *c*, which ensures that the apoptotic decision is irreversible [14,15].

The extrinsic apoptotic pathways, also known as ‘death receptor pathways’, are initiated through the activation of membrane death receptors, including Fas, TNFR, DR3 and DR4/DR5, by their respective ligands (FasL, TNF *α*, TWEAL and Trail) [16,17]. Engagement of death receptors by their cognate ligands triggers the recruitment of different adaptor proteins. Depending on the recruited adaptors, either pro-apoptotic signals (mediated by caspases-8 and -10) or pro-survival (MAPK- and/or NF- *κ*B-mediated) signals are induced. Caspase-8 and -10 recruited to death receptors autoactivate themselves and activate further effector caspases (caspase-3 and -7) either by direct processing or by engaging the intrinsic death pathway [11].

The intrinsic apoptotic pathway is also called the mitochondrial pathway because it is associated with the disruption of mitochondrial outer membranes and consequent release of cytochrome *c*. This process is regulated by the Bcl-2 family proteins, which share up to four BH (Bcl-2 homology) domains. The proteins can be classified as: pro-apoptotic multidomain effectors (Bax and Bak), pro-survival multidomain restrainers (including i.a. Bcl-2 proper, Bcl-x_L_, Bcl-w, Mcl-1, A1) and pro-apoptotic single domain BH3-only upstream sentinels (comprising Bid, Bim, Bad, Puma, Noxa and others) [18,19].

Bax and Bak are effectors directly responsible for the mitochondrial outer membrane permeabilization via either channel formation [20] or opening of voltage-dependent anion channels [21]. While these effectors are present even in surviving cells, activated p53 can induce transcription of their genes and further elevate levels of both Bax [22] and Bak [23]. Deletion of either *bax* or *bak* affects apoptosis only slightly, but deletion of both these genes dramatically impairs apoptosis in many tissues [24,25]. Despite intense studies, it is still controversial, how the level, conformation and activity of these pro-apoptotic effectors is regulated. There is a bulk of evidence that Bax and Bak (plausibly after initial conformational priming by BH3-only proteins [26,27]) can be inhibited by pro-survival restrainers [28-30] (either by direct sequestration in the mitochondrial membrane [31] or by active retrotranslocation to the cytosol [32,33]). Other studies suggest that Bax and Bak could be activated directly by some BH3-only proteins (Bid, Bim, Puma) [26,27,34,35], however *bim*^–/–^*bid*^–/–^ mice develop rather normally [36]. Additionally, the release of Bax from pro-survival restrainers appears to be sufficient to trigger apoptosis [37], and these interactions have been characterized in detail recently [38,39]. The function of another subset of BH3-only proteins (Bad, Bid, Noxa) is proposed to rely mainly on displacing (conformationally primed) pro-apoptotic effectors from pro-survival restrainers [40,41]. This mechanism is enabled by much higher affinity of BH3-only proteins to restrainers in comparison to effectors: in cells with abundant Bcl-x_L_:Bad heterodimers, no Bax:Bcl-x_L_ heterodimers are present [31,42]. In normal cells Bad is found mostly in the phosphorylated form (at Ser112 and Ser136 by phosphorylated Akt), in which it preferentially binds to the scaffolding protein 14-3-3 (isoform *θ*) instead of Bcl-2 family pro-survival restrainers [43-45]. Although direct interaction of Bax and 14-3-3 has been demonstrated and overexpression of 14-3-3 inhibits apoptosis, prior caspase activity is required for cleaving 14-3-3 to release Bax [46].

Bcl-2 family proteins are activated or inhibited in response to numerous stress factors including heat shock, *γ* and UV irradiation, nutrient deprivation, viral infection, hypoxia and increased intracellular calcium concentration [47,48]. In this study, we confine to two sources of external stimuli: 1) DNA damage prompting the activation of p53 (which mediates pro-apoptotic signals) [49,50] and 2) withdrawal of growth factors (GF) leading to the deactivation of Akt (which, when active, mediates anti-apoptotic signals) [51-53].

In not onco-transformed cells, p53 protein remains inactive [54]. In response to DNA damage, p53 is activated by phosphorylation at Ser15 and Ser20 (by ATM [55,56]), which protects it from rapid degradation [56,57]. When phosphorylated, p53 is capable of inducing synthesis of its own inhibitors: ubiquitin-protein ligase Mdm2 and serine/threonine phosphatase Wip1 [58,59], as well as proteins responsible for cell cycle arrest and DNA repair [60]. Additional p53 phosphorylation at Ser46 (by kinase HIPK2 [61,62]) enables p53 to activate expression of proteins which mediate apoptosis [63], in particular pro-apoptotic Bax and Bak [22,23]. DNA repair and apoptotic functions make p53 a primary tumor suppressor; respectively the p53 gene is the most frequently mutated gene in cancers [64,65].

In healthy cells but also frequently in cancer cells, Akt, in contrast to p53, maintains its (at least partial) activity and suppresses apoptotic signals by phosphorylating and thereby inhibiting pro-apoptotic Bad [66]. Akt activity is controlled by growth factors, which stimulate membrane receptors and induce activation of Ras, transmitting signal to PI3K, which in turn phosphorylates PIP2 into PIP3 [51,67] (PI3K can respond to growth factors also independently of Ras [68]). PIP3 enables membrane localization of Akt, allowing for Akt activation via phosphorylation at Thr308 and Ser473 by kinase PDK1 [69]. The anti-apoptotic Akt and its upstream regulators, such as GTPase Ras and kinase PI3K, are deregulated in a wide range of solid tumors and hematologic malignancies, hence the Akt pathway is considered the key determinant of biological aggressiveness of these tumors and a major potential target for anticancer therapies [70,71].

Interestingly, phosphorylation of p53 at Ser46 enables it to activate expression of phosphatase PTEN [72,73], which prevents phosphorylation of Akt by dephosphorylating PIP3 to PIP2. Only if activated, Akt mediates phosphorylation of the p53 primary inhibitor, Mdm2, allowing it to localize to the nucleus and prime p53 for degradation [74,75]. These interactions intertwine tightly signaling of pro-apoptotic p53 and anti-apoptotic Akt.

### Apoptotic models

Here we review mathematical models of the apoptotic pathway, which are relevant to our study.

Stucki and Simon [76] have focused on inhibitors of apoptosis (IAPs) that are able to bind active caspases leading to their degradation in the proteasome. They proposed a simple mathematical model, describing the molecular interactions between Smac, Smac deactivators, IAPs, and caspase-3, and derive the requirements for either induction or prevention of apoptosis, which is initiated when the level of caspase-3 exceeds a given threshold. Further, Bagci *et al*. [77] described a mathematical representation of mitochondria-dependent apoptosis, in which kinetic cooperativity in the formation of the apoptosome (which consists of 7 cytochrome *c*–Apaf dimers) is a key element ensuring bistability in survival-or-death decisions. They examined the influence of Bax and Bcl-2 synthesis and degradation rates, as well as the number of mitochondrial permeability transition pores (MPTPs) on the cell response to apoptotic stimuli. The model predicts that above some critical Bax degradation rate the system is monostable and cells survive. Bistability arises for Bax degradation smaller than critical, while the cell fate depends on the initial level of caspase-3. When the number of MPTPs is large, the bistability vanishes and the apoptosis is initiated regardless of the initial condition.

Wee *et al*. [78] analyzed the mutual antagonism between pro-apoptotic signals of p53 and pro-survival signals of Akt in response to DNA damage. The coupling between p53 and Akt involves p53-regulated PTEN which dephosphorylates PIP3 required for Akt phosphorylation. The considered apoptotic module comprises four proteins from the Bcl-2 family: pro-apoptotic Bax and Bad (transcription of which is triggered by p53) and anti-apoptotic Bcl-2 and Bcl-x_L_ (binding, respectively, Bax and Bad). In the model the pro-survival action of Akt relies on Bad phosphorylation which primes it for degradation. The authors demonstrated that repeated oscillations of the p53 level lead to the depletion of Bcl-2 and Bcl-x_L_, which is considered as a marker of apoptosis. Antagonism between pro-apoptotic signals of p53 and pro-survival signals of Akt can also lead to bistability, which, if DNA repair is not accomplished in time, allows for termination of p53 oscillations [79-81], significant increase of the p53 level and eventual apoptosis [82,83]. In correspondence to the mentioned studies [78,82,83], Li *et al*. [84] constructed an integrated mathematical model that includes three modules of the p53 network: p53 core regulation, p53-induced cell cycle arrest and p53-dependent apoptosis initiation. Analysis of the model reveals that different aspects of the nuclear p53 dynamic profile are used to differentially regulate the pro-survival and pro-apoptotic modules. The initiation of caspase activity happens only when both the nuclear and the mitochondrial p53 levels are above certain thresholds. However, the switch to the apoptotic state is mainly triggered by the accumulation of the mitochondrial p53, which proceeds during oscillations of nuclear p53. Further, Tian *et al*. [85] proposed a two step mechanism coordinating (early) mitochondrial and (late) nuclear p53 activities. In the model, apoptosis may quickly follow the severe DNA damage through the mitochondrial p53 pathway, or, in the case of less severe damage, apoptosis may follow the cycle arrest phase if DNA repair has not been accomplished.

In summary, the above models revealed that apoptosis is possible based on the system bistability, which makes the apoptotic decision irreversible. Bagci *et al*. [77] showed that bistability may arise due to cooperativity in the apoptosome formation and two positive feedback loops mediated by caspase-3. In the model of Tian *et al*. [85] bistability arises due to cooperation of two positive feedbacks: one is the double-negative feedback loop between p21 and caspase-3, the other positive feedback loop is between cytochrome *c* and caspase-3. In correspondence to the model proposed here, the study of Wee *et al*. [78] indicated that the apoptotic decision is controlled by opposing pro- and anti-apoptotic signals mediated respectively by p53 and Akt. Finally, Schlatter *et al*. [86] presented an apoptotic model based on the time-resolved Boolean network with multi-value node logic, which allowed them to emulate typical apoptotic features. The model is analyzed with regard to its internal connectivity and crosstalks, with special attention to feedback loops and delayed processes such as gene regulation.

**Figure 1:**
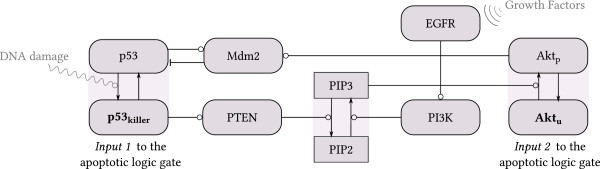
**Crosstalk of pro-apoptotic and pro-survival pathways.** The intracellular pro-apoptotic signal is mediated through p53_killer_. Elevated *p*53_killer_ activates expression of PTEN, which leads to the inactivation of pro-survival Akt. Activity of Akt is also regulated by extracellular growth factors. For the apoptotic module analysis, both input signals, *p*53_killer_ and *Akt*_u_, are considered independently. Arrow-headed lines indicate protein transformation, circle-headed lines denote activation or transcriptional regulation, hammer-headed lines denote repression.

In this study we aim to address the question, *whether the interaction network of Bcl-2 family proteins allows for the integration of apoptotic signals, and if yes, whether this signal integration is analogous to Boolean logic gates*. Boolean networks are used to model various regulatory pathways, including apoptotic ones [86], however, the analysis of correspondence between biochemical reaction kinetics (which can be approximated by systems of ODEs) and the Boolean approach is missing. Here, following Wee *et al*. [78] we focus on two key pathways leading to apoptosis: one mediated by p53 and the other by Akt. As already discussed, p53 activates expression of PTEN, thus the elevated level of p53 results in the decreased level of phosphorylated Akt. However, since Akt activity is also regulated by growth factors (Figure 1), in our model the p53 and Akt pathways will be considered independently. We will analyze how the apoptotic signals are collected and processed before the apoptosis-or-survival decision is attained. In accordance with previous studies, we will assume that the apoptotic switch is based on bistability arising due to positive feedback mediated by caspase-3, and nonlinearity. We will demonstrate that the topology of the mitochondrial apoptotic module allows for the integration of signals in the manner analogous to the logic gates AND or OR, depending on the levels of pro-apoptotic Bad and pro-survival Bcl-x_L_.

## Methods

The system of ODEs following the proposed model was solved using the ode23tb solver implemented in MATLAB (The MathWorks Inc., Natick, MA, USA). The source code is available as the Additional file 1.

## Results

### Model

Our model of the apoptotic decision module involves the following components: p53, Akt, scaffolding protein 14-3-3, caspases, and the Bcl-2 family proteins. The evidence that p53 serves as a transcription factor for *bax* is well-established [22]. Contrary to Bak, Bax can interact with any pro-survival restrainer [36]. The competition for Bcl-x_L_ was reported between Bax and Bad, with the latter having higher affinity [42]. For these reasons, pro-apoptotic effectors Bax and Bak are represented collectively by a single entity termed Bax, and Bcl-x_L_ is selected to represent the group of restrainers. Neutralization of Bcl-x_L_ is the primary pro-apoptotic function of Bad [87,88], and it was demonstrated that a single mutation in Bad is sufficient to disrupt Bad:Bcl-2 but not Bad:Bcl-x_L_ binding [88]; Bad binds to restrainers stronger than e.g. Bid [40]. Thus, BH3-only proteins are represented by a single Bad.

Active Bax, when freed from its antagonists, releases cytochrome *c* from the mitochondrial intermembrane space [28]. Released cytochrome *c* enables the formation of the apoptosome, which in turn initiates the downstream caspase program [10]. In the model, we omit these steps and assume that free Bax induces cleavage of pro-caspases into active caspases. The major caspase involved in this step is caspase-9, which can cleave itself into the active form within the apoptosome, and then activates the main executor caspase-3. Caspase-3 cleaves Bcl-2 leading to the further release of cytochrome *c* creating positive feedback loop, which introduces bistability and makes the apoptotic decision irreversible [14,15,83]. In the model, for the sake of simplicity, we consider a single caspase species and we simplify the positive feedback loop to the caspase auto-activation mechanism.

**Figure 2:**
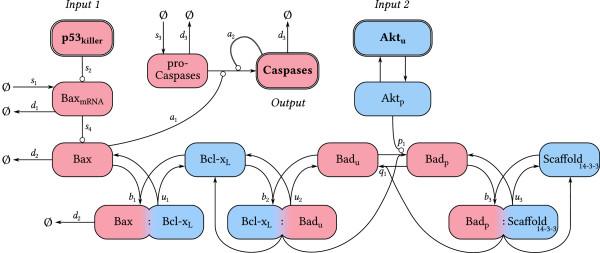
**Mitochondrial apoptotic module.** The levels of p53_killer_ and Akt_u_ are considered as pro-apoptotic input signals. Total level of Akt is assumed constant and, since the phosphorylated Akt is anti-apoptotic, the unphosphorylated Akt is considered as the pro-apoptotic input (associated with e.g. growth factor withdrawal). Phosphorylated Akt phosphorylates Bad, which then remains bound to Scaffold_14-3-3_. When dephosphorylated, Bad binds to Bcl-x_L_, leading to the dissociation of Bax from its complexes with Bcl-x_L_. Free Bax (via the omitted process of cytochrome *c* release) turns pro-caspases into active caspases (module output) which induce apoptosis. Arrow-headed lines indicate protein transformations, circle-headed lines – activating (including transcription) or inactivating influence. Light red color indicates pro-apoptotic, and light blue – anti-apoptotic proteins. Symbol Ø is used to denote sink or source.

In the model (Figure 2), levels of phosphorylated Akt (named here Akt_p_) and p53 in its killer form (i.e. p53 phosphorylated at Ser15, Ser20 and Ser46; denoted p53_killer_) will serve as inputs. Surviving cells are characterized by high level of Akt_p_ and lack (or very low level) of p53_killer_. In these cells, most of Bad remains in the phosphorylated form (denoted Bad_p_) bound to Scaffold_14-3-3_, while Bax is inhibited (sequestered) by Bcl-x_L_[44]. Unphosphorylated Bad (Bad_u_) may bind to Bcl-x_L_, which limits the amount of Bcl-x_L_ protein available for inhibiting Bax. The steady state is controlled by the total levels of Bad (*Bad*_tot_) and Bcl-x_L_ (*Bcl*-*x*_*L* tot_), which will be considered as parameters in the model. The fraction of Bcl-x_L_ bound to Bad increases with *Bad*_tot_ rendering cells of high Bad level more prone to apoptosis. (Protein levels, written in italics, are given in molecules per cell; for disambiguation, levels of protein complexes will be written in curly braces.)

We consider two pro-apoptotic stimuli, which may lead to the accumulation of free Bax and consequent apoptosis: 

1) increase of the level of p53_killer_, which triggers Bax transcription and Bax protein accumulation;

2) dephosphorylation of Akt, i.e. decrease of *Akt*_p_ and corresponding increase of the level of unphosphorylated Akt (*Akt*_u_).

Akt dephosphorylation is followed by the dephosphorylation of Bad and its release from Scaffold_14-3-3_. Dephosphorylated Bad captures Bcl-x_L_ which eventually releases Bax.

*Important notice*: We assume that the total level of Akt (*Akt*_tot_=*Akt*_p_+*Akt*_u_) remains constant. Under this assumption, the appearance of unphosphosphorylated Akt (Akt_u_) will be considered as a pro-apoptotic stimulus. The strength of both pro-apoptotic stimuli will be thus measured by *p*53_killer_ and *Akt*_u_.

The mathematical representation of the apoptotic module consists of 11 ordinary differential equations for levels of molecular species or their complexes: Bax_mRNA_, Bax, Bcl-x_L_, Bax:Bcl-x_L_, Bcl-x_L_:Bad_u_, Bad_u_, Bad_p_, Bad_p_:Scaffold_14-3-3_, Scaffold_14-3-3_, pro-caspases and caspases.

$$\begin{array}{lll} \;\frac{d}{dt}{\text{Bax}}_{\text{mRNA}}(t) & =s_{1}+s_{2}\frac{{p{\text{53}}_{\text{killer}}}^{2}}{M^{2}+{p{\text{53}}_{\text{killer}}}^{2}}\; & \;\\ & \;-d_{1}{\text{Bax}}_{\text{mRNA}},\; & \;\\ \;\frac{d}{dt}\text{Bax}(t) & =s_{4}{\text{Bax}}_{\text{mRNA}}+u_{1}\{\text{Bax}:\;\text{Bcl}\text{-}x_{\;L}\}\; & \;\\ & \;-b_{1}\text{Bax}\;\cdot \;\text{Bcl}\text{-}x_{\;L}-d_{2}\text{Bax},\; & \;\\ \;\frac{d}{dt}\text{Bcl}\text{-}x_{\;L}(t) & =u_{2}\{\text{Bcl}\text{-}x_{\;L}:\;{\text{Bad}}_{u}\}+u_{1}\{\text{Bax}:\;\text{Bcl}\text{-}x_{\;L}\}\; & \;\\ & \;+p_{1}{\text{Akt}}_{p}\{\text{Bcl}\text{-}x_{\;L}:\;{\text{Bad}}_{u}\}\; & \;\\ & \;-b_{2}\text{Bcl}\text{-}x_{\;L}\;\cdot \;{\text{Bad}}_{u}-b_{1}\text{Bax}\;\cdot \;\text{Bcl}\text{-}x_{\;L}\; & \;\\ & \;+d_{2}\{\text{Bax}:\;\text{Bcl}\text{-}x_{\;L}\},\; & \;\\ \;\frac{d}{dt}\{\text{Bax}:\;\text{Bcl}\text{-}x_{\;L}\}(t) & =b_{1}\text{Bax}\;\cdot \;\text{Bcl}\text{-}x_{\;L}-u_{1}\{\text{Bax}:\;\text{Bcl}\text{-}x_{\;L}\}\; & \;\\ & \;-d_{2}\{\text{Bax}:\;\text{Bcl}\text{-}x_{\;L}\},\; & \;\\ \;{\text{Bad}}_{u}\}(t) & =b_{2}\text{Bcl}\text{-}x_{\;L}\;\cdot \;{\text{Bad}}_{u}-u_{2}\{\text{Bcl}\text{-}x_{\;L}:\;{\text{Bad}}_{u}\}\; & \;\\ & \;-p_{1}{\text{Akt}}_{p}\{\text{Bcl}\text{-}x_{\;L}:\;{\text{Bad}}_{u}\},\; & \;\\ \;\frac{d}{dt}{\text{Bad}}_{u}(t) & \;=\;u_{2}\{\text{Bcl}\text{-}x_{\;L}:\;{\text{Bad}}_{u}\}-b_{2}\text{Bcl}\text{-}x_{\;L}\;\cdot \;{\text{Bad}}_{u}\; & \;\\ & \;-p_{1}{\text{Akt}}_{p}\;\cdot \;{\text{Bad}}_{u}+q_{1}{\text{Bad}}_{p}\; & \;\\ & \;+q_{1}\{{\text{Bad}}_{p}:\;{\text{Scaffold}}_{14-3-3}\},\; & \;\\ \;\frac{d}{dt}{\text{Bad}}_{p}(t) & =u_{3}\{{\text{Bad}}_{p}:\;\text{Scaffold}14-3-3\}\; & \;\\ & \;-b_{3}{\text{Bad}}_{p}\;\cdot \;{\text{Scaffold}}_{14-3-3}\; & \;\\ & \;+p_{1}{\text{Akt}}_{p}\;\cdot \;{\text{Bad}}_{u}\; & \;\\ & \;+\;p_{1}{\text{Akt}}_{p}\{\text{Bcl}\text{-}x_{\;L}:\;{\text{Bad}}_{u}\}\;-\;q_{1}{\text{Bad}}_{p},\; & \;\\ \;\frac{d}{dt}\{{\text{Bad}}_{p}:\;{\text{Scaffold}}_{14-3-3}\}(t) & =b_{3}{\text{Bad}}_{p}\;\cdot \;{\text{Scaffold}}_{14-3-3}\; & \;\\ & \;-u_{3}\{{\text{Bad}}_{p}:\;{\text{Scaffold}}_{14-3-3}\}\; & \;\\ & \;-q_{1}\{{\text{Bad}}_{p}:\;{\text{Scaffold}}_{14-3-3}\},\; & \;\\ \;\frac{d}{dt}{\text{Scaffold}}_{14-3-3}(t) & =u_{3}\{{\text{Bad}}_{p}:\;{\text{Scaffold}}_{14-3-3}\}\; & \;\\ & \;-b_{3}{\text{Bad}}_{p}\;\cdot \;{\text{Scaffold}}_{14-3-3}\; & \;\\ & \;+q_{1}\{{\text{Bad}}_{p}:\;{\text{Scaffold}}_{14-3-3}\},\; & \;\\ \;\frac{d}{dt}\text{Procasp}(t) & =s_{3}-a_{1}\text{Bax}\;\cdot \;\text{Procasp}\; & \;\\ & \;-a_{2}{\text{Casp}}^{2}\;\cdot \;\text{Procasp}\; & \;\\ & \;-d_{3}\text{Procasp},\; & \;\\ \;\frac{d}{dt}\text{Casp}(t) & =a_{1}\text{Bax}\;\cdot \;\text{Procasp}\; & \;\\ & \;+a_{2}{\text{Casp}}^{2}\;\cdot \;\text{Procasp}\; & \;\\ & \;-d_{3}\text{Casp}.\; & \; \end{array}$$

Bax_mRNA_ transcription, induced by p53 _killer_, is modeled according to the sigmoidal kinetics (Hill equation with cooperativity coefficient equal 2) [89]. The cubic term in caspase activation introduces bistability and assures irreversibility of the apoptotic decision [15]. Model parameters are provided in Table 1.

**Table 1:** Model parameters

***Symbol***	***Value***	***Unit***	***Description***
*s*_1_	10^−2^	mlcl × s^−1^	Basal Bax_mRNA_ synthesis rate
*s*_2_	3 × 10^−2^	mlcl × s^−1^	p53_killer_-regulated Bax_mRNA_ synthesis rate
*s*_3_	2 × 10^1^	mlcl × s^−1^	Pro-caspases synthesis rate
*s*_4_	2 × 10^−1^	s^−1^	Bax protein synthesis
*M*	10^5^	mlcl	Michaelis–Menten coefficient for p53_killer_-regulated Bax transcription
*d*_1_	10^−3^	s^−1^	Bax_mRNA_ degradation rate
*d*_2_	10^−4^	s^−1^	Bax degradation rate
*d*_3_	2 × 10^−4^	s^−1^	Pro-caspases and caspases degradation rate
*b*_1_	3 × 10^−5^	mlcl^−1^s^−1^	Bax–Bcl-x_L_ binding rate
*b*_2_	3 × 10^−3^	mlcl^−1^s^−1^	Bcl-x_L_–Bad_u_ binding rate
*b*_3_	3 × 10^−3^	mlcl^−1^s^−1^	Bad_p_–Scaffold_14-3-3_ binding rate
*u*_1_	10^−4^	s^−1^	Bax:Bcl-x_L_ heterodimer unbinding rate
*u*_2_	10^−4^	s^−1^	Bcl-x_L_:Bad_u_ heterodimer unbinding rate
*u*_3_	10^−4^	s^−1^	Bad_p_:Scaffold_14-3-3_ heterodimer unbinding rate
*p*_1_	3 × 10^−10^	s^−1^	Bad_u_ phosphorylation rate in Bcl-x_L_:Bad_u_ heterodimer (by Akt_p_)
*q*_1_	3 × 10^−5^	s^−1^	Bad_p_ dephosphorylation rate
*a*_1_	2 × 10^−10^	mlcl^−1^s^−1^	Pro-caspases activation rate (by Bax)
*a*_2_	10^−12^	mlc*l*^−2^s^−1^	Pro-caspases autoactivation rate
*Akt*_tot_	2 × 10^5^	mlcl	Constant pool of Akt, *Akt*_tot_=*Akt*_u_+*Akt*_p_
$p53_{\text{killer}}^{\text{max}}$	2 × 10^5^	mlcl	Maximum allowed level of *p*53_killer_

Abbreviation ‘mlcl’ denotes the number of molecules per cell.

### The caspase switch

**Figure 3:**
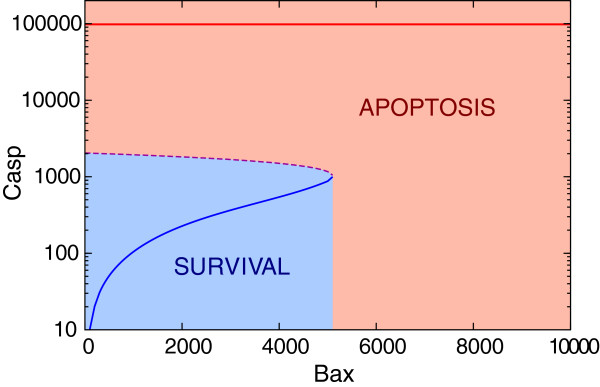
**Bifurcation diagram for *Casp* vs. *Bax*.** Saddle-node bifurcation point is (*Bax*_bif_, *Casp*_bif_) ≃ (5000, 1000). The unstable steady state is marked by dashed line. Solid lines show high and low stable steady states corresponding to apoptosis and survival, respectively. (Note the logarithmic scale on the vertical axis).

The last two equations exhibit bistability. In Figure 3 we show the bifurcation diagram of *Casp* with *Bax* considered as a bifurcation parameter. *Casp* undergoes the saddle-node bifurcation at *Bax*=*Bax*_bif_≃5000 (with *Casp*≃1000). Below the bifurcation point there exist three steady states: one unstable and two stable corresponding to low (<1000) and high (>9.9 × 10^4^) caspase levels. Above the bifurcation point there exists the unique stable steady state characterized by high *Casp*. The structure of the bifurcation diagram ensures that the apoptotic switch is irreversible, i.e. once the system switches to the apoptotic state, it may not switch back even if the level of free effectors, *Bax*, drops to zero. The apoptotic switch occurs when *Bax* exceeds *Bax*_bif_ for sufficiently long time; as we will show later, short excursions of *Bax* over *Bax*_bif_ do not induce apoptosis. For the steady state analysis, however, the states with *Bax*>*Bax*_bif_ will be interpreted as apoptotic.

### OR and AND logic gates

We demonstrate that apoptosis can be controlled in a manner similar to logic gates OR and AND with inputs defined as *p*53_killer_ and *Akt*_u_. In this section, we consider digital steady-state inputs: the signal from p53_killer_ is assumed to be logic **0** for *p*53_killer_=0, and is assumed logic **1** for $p53_{\text{killer}}=p53_{\text{killer}}^{\text{max}}=2\times 10^{5}$, which is the highest level considered in the analysis. The signal from the Akt branch is interpreted as logic **0** for *Akt*_u_=0 (i.e. when all Akt is in the phosphorylated form) and is considered logic **1** when *Akt*_u_=*Akt*_tot_=2 × 10^5^. Intermediate input values of $p53_{\text{killer}}\in (0,\;p53_{\text{killer}}^{\text{max}})$ and *Akt*_u_∈(0, *Akt*_tot_) will be analyzed in a forthcoming section.

**Figure 4:**
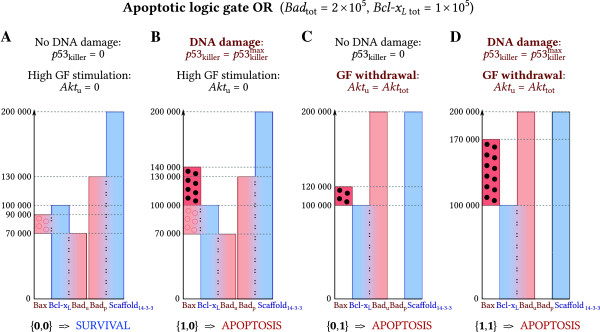
**Steady-state concentrations for gate OR.** The presence of a single sufficiently strong input signal, *p*53_killer_ or *Akt*_u_, triggers apoptosis. Heights of bars correspond to the steady-state levels of proteins. Bars sharing adjacent sides (marked with colons and color gradient) denote complexes. To keep the convention, some bars do not start from 0. Accordingly, in **(A)** there are about 2 × 10^4^ molecules of Bax:Bcl-x_L_ complex, about 1 × 10^4^ molecules of free Bcl-x_L_, about 7 × 10^4^ molecules of Bcl-x_L_:Bad_u_ complex, about 13 × 10^4^ Bad_p_:Scaffold_14-3-3_ complex, and about 7 × 10^4^ molecules of free Scaffold_14-3-3_. **(A)** The steady state levels of proteins and complexes corresponding to *p*53_killer_ = 0 and *Akt*_u_ = 0 (surviving cell, in the presence of growth factors). In order to induce apoptosis, gate OR requires a single sufficiently strong signal from **(B)** p53_killer_ (*p*53_killer_ = 2 × 10^5^) or **(C)** Akt_u_ (*Akt*_u_ = 2 × 10^5^). **(D)** Simultaneous presence of both input signals (*p*53_killer_ = 2 × 10^5^, *Akt*_u_ = 2 × 10^5^) results in higher level of Bax than in the case of stimulation by a single signal.

**Figure 5:**
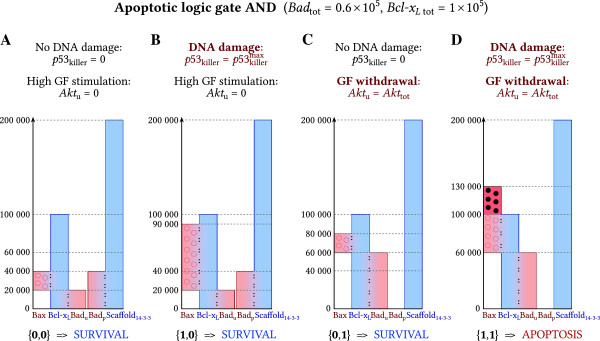
**Steady-state concentrations for gate AND.** Transition from OR to AND results from the decrease of *Bad*_**tot**_. The simultaneous presence of both input signals, *p53*_killer_ and *Akt*_u_, is necessary to induce apoptosis. Heights of bars correspond to the steady-state levels of proteins. Bars sharing adjacent sides (marked with colons and color gradient) denote complexes. To keep the convention, some bars do not start from 0. **(A)** The steady state levels of proteins and complexes corresponding to *p53*_killer_* = 0* and *Akt*_u_* = 0* (surviving cell, in the presence of growth factors). **(B, C)** a single maximal pro-apoptotic signal neither from p53_killer_ nor from Akt_u_ suffices to trigger apoptosis. (D) Simultaneous presence of both signals triggers apoptosis.

We will analyze the steady states of the system for two levels of Bad in order to demonstrate that the gate OR is achieved for *Bad*_tot_=2 × 10^5^, while gate AND is achieved for *Bad*_tot_=0.6 × 10^5^; here, for both gates we assume the same *Bcl*-*x*_*L* tot_=1 × 10^5^. Surviving cell steady state is associated with conditions in which both apoptotic signals are equal zero. In these cells (Figures 4A and 5A) most of Bad is in the phosphorylated form bound to Scaffold_14-3-3_. The remaining (unphosphorylated) Bad is bound to Bcl-x_L_. Rest of Bcl-x_L_ sequesters Bax or remains free. Free Bcl-x_L_ may be considered as the anti-apoptotic buffer, which may potentially capture the excess of Bax. This buffer of free Bcl-x_L_ is small (about 10^4^ molecules) for gate OR (Figure 4A) and much larger (about 6 × 10^4^ molecules) for gate AND (Figure 5A). Size of the Bcl-x_L_ buffer determines cell susceptibility to apoptosis: small buffer renders cells more prone to apoptosis.

Accordingly, to trigger apoptosis, gate OR (Figure 4) requires any of two signals. The signal coming from p53 (Figure 4B) causes that the total Bax level increases such that *Bax*_tot_*>Bcl-x*_*L tot*_*−Bad*_u_. As a result, not all Bax may be sequestered by Bcl-x_L_ and free Bax appears. The signal coming from Akt (whole Akt desphosphorylation, Figure 4C) causes dephosphorylation of Bad, which is released from Scaffold_14-3-3_ and captures the whole pool of Bcl-x_L_. As a result, all Bax (2 × 10^4^ molecules) is released.

Gate AND arises when the total level of Bad is smaller and correspondingly the initial (surviving cell steady state) level of (free) Bcl-x_L_ is larger (Figure 5, Table 2). In this case, both pro-apoptotic signals are required to trigger apoptosis. Although the p53 signal increases the level of Bax, still *Bax*_tot_*<Bcl-x*_*L* tot_*−Bad*_u_, and thus all Bax remains sequestered by Bcl-x_L_ (Figure 5B). The signal coming from the Akt branch leads to dephosphorylation of Bad, which dissociates from Scaffold_14-3-3_ and binds to Bcl-x_L_ (Figure 5C). However, again *Bax*_tot_*** < Bcl-x***_*L* tot_***−Bad***_u_ and thus all Bax remains captured by Bcl-x_L_. Only the combination of signals coming from p53 and Akt, which leads to the simultaneous increase of the total Bax level and partial sequestration of Bcl-x_L_ by Bad_u_, results in the release of Bax (since *Bax*_tot_* > Bcl-x*_*L* tot_*−Bad*_u_) and eventually triggers apoptosis (Figure 5D).

**Table 2:** Apoptotic gate types resulting from considered Bcl-2 family protein levels

***Protein levels***			***Gate type***
***Bax***_**tot**_	***Bcl-x***_***L* tot**_	***Bad***_**tot**_	
dynamic	***1 × 10***^*5*^	*2 × 10*^*5*^	OR
dynamic	*1 × 10*^*5*^	*0.6 × 10*^**5**^	AND
dynamic	*2.4 × 10*^**5**^	*2 × 10*^*5*^	AND*

Levels of total Bcl-x_L_ and Bad are assumed constant while the level of total Bax is dynamically regulated.

**Figure 6:**
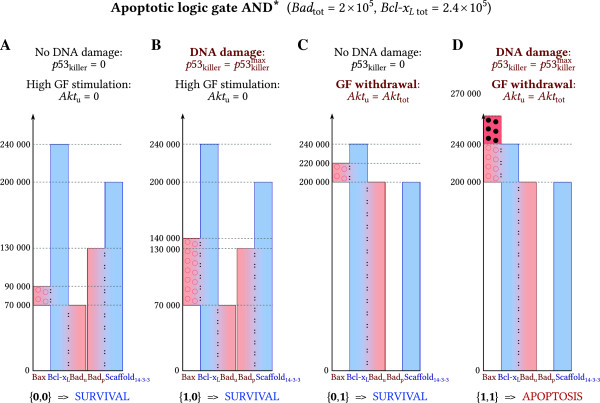
Steady-state concentrations for gate AND*. Transition from OR to AND* results from the increase of *Bcl-x*_*L* tot_. The simultaneous presence of both input signals,*p53*_killer_ and *Akt*_u_, is necessary to induce apoptosis. Heights of bars correspond to the steady state levels of proteins. Bars sharing adjacent sides (marked with colons and color gradient) denote complexes. To keep the convention, some bars do not start from 0. **(A)** The steady state levels of proteins and complexes corresponding to *p53*_killer_* = 0* and *Akt*_u_* = 0* (surviving cell, in the presence of growth factors). **(B, C)** A single maximal pro-apoptotic signal neither from p53_killer_ nor from Akt_u_ suffices to trigger apoptosis. **(D)** Simultaneous presence of both signals triggers apoptosis.

Alternatively, gate OR may be transformed into gate AND (denoted AND* to avoid confusion) by a significant increase of the level of pro-survival Bcl-x_L_ (Figure 6, Table 2). When*Bcl-x*_*L* tot_*=2.4 × 10*^*5*^ (with *Bad*_tot_*=2 × 10*^*5*^), dephosphorylated Bad is not able to displace Bax from its complex with Bcl-x_L_: high abundance of Bcl-x_L_ allows it to restrain whole Bad_u_ and whole Bax at the same time.

In the next section we summarize the above numerical analysis introducing approximate formulas defining OR and AND gates.

### Macro-parameters characterizing steady-state solutions

An important property of the model is that, as long as steady-state solutions are considered, a change of any of its 20 parameters can be compensated by a proper change of other (conjugate) parameter(s). Therefore, steady-state concentrations of proteins depend on clusters of parameters rather than on individual parameter values. We identified 7 such parameter clusters, which define ‘macro-parameters’ (Table 3). When considered pairwise, macro-parameters are mostly mutually independent.

**Table 3:** Macro-parameters governing steady-state solution levels

***Macro-parameter definition***	***Value***	***Unit***	***Description***
$m_{1}:=\frac{s_{1}s_{4}}{d_{1}d_{2}}$	*2 × 10*^*4*^	mlcl	${\text{Bax}}_{\text{tot}}^{\text{min}}$
$m_{2}:=\left(s_{1}+s_{2}\frac{{\left(p53_{\text{killer}}^{\text{max}}\right)}^{2}}{M^{2}+{\left(p53_{\text{killer}}^{\text{max}}\right)}^{2}}\right)\frac{s_{4}}{d_{1}d_{2}}$	*6.8 × 10*^*4*^	mlcl	${\text{Bax}}_{\text{tot}}^{\text{max}}$
$m_{3}:=\frac{b_{1}}{u_{1}}$	0.3	mlcl^*−1*^	*Bax–Bcl-x*_*L*_ affinity
$m_{4}:=\frac{b_{2}}{u_{2}}$	30	mlcl^*−1*^	*Bcl-x*_*L*_–*Bad*_u_ affinity
$m_{5}:=\frac{b_{3}}{u_{3}}$	30	mlcl^*−1*^	*Bad*_p_–*Scaffold*_14-3-3_ affinity
$m_{6}:=\frac{p_{1}\text{Ak}t_{\text{tot}}}{q_{1}+p_{1}\text{Ak}t_{\text{tot}}}$	$\frac{2}{3}$	—	max. fraction of phosphorylated Bad
$m_{7}:=\frac{d_{3}S-a_{2}S^{2}(\frac{s_{3}}{d_{3}}-S)}{a_{1}(\frac{s_{3}}{d_{3}}-S)}$,	≅ 5000	mlcl	*Bax*_bif_ (at *Casp*≅ 1000)
where*S*=*Casp*+*Procasp*			

Abbreviation ‘mlcl’ denotes the number of molecules per cell.

Based on the macro-parameters one can obtain approximate algebraic formulas which must be satisfied for a given gate type. First, let us notice that since all (non-dimensional) dissociation constants *1/m*_3_, *1/m*_4_, and *1/m*_5_ are larger than the characteristic numbers of molecules, all allowed complexes are formed. The condition*m*_3_≪*m*_4_ implies that Bcl-x_L_ binds preferentially to Bad_u_ and therefore free Bax is released only when

$${\text{Bax}}_{\text{tot}}+{\text{Bad}}_{u}>\text{Bcl}\text{-}x_{L}.$$

When the level of free Bax exceeds*Bax*_bif_, the cell sets in the apoptotic state; in the opposite case the cell survives. The amount of Bad_u_ is (1−*m*_6_)*Bad*_tot_. In this way we obtain simple inequalities defining logic gates. These inequalities, given in Tables 4 and 5, show the connection between ODEs and Boolean logic gates, and pose restrains on parameter values.

**Table 4:** Algebraic conditions for outputs of the OR gate

**Gate OR**		
***Inputs***	***Output***	***Condition for the output***
{**0**,**0**}	Survival	*m*_1_+(1−*m*_6_)*Bad*_tot_<*Bcl-x*_*L*_+*m*_7_
{**1**,**0**}	Apoptosis	*m*_2_+(1−*m*_6_)*Bad*_tot_>*Bcl-x*_*L*_+*m*_7_
{**0**,**1**}	Apoptosis	*m*_1_+*Bad*_tot_>*Bcl-x*_*L*_+*m*_7_
{**1**,**1**}	Apoptosis	*m*_2_+*Bad*_tot_>*Bcl-x*_*L*_+*m*_7_

Input in the format {*i,j*} denotes *p*53_killer_ =*i* × 2 × 10^5^,*Akt*_u_ =*j* × 2 × 10^5^. It is assumed that*m*_3_ ≪*m*_4_.

**Table 5:** Algebraic conditions for outputs of the AND and AND* gates

Gates AND and AND*		
***Inputs***	***Output***	***Condition for the output***
{**0**,**0**}	Survival	*m*_1_+(1−*m*_6_)*Bad*_tot_<*Bcl-x*_*L*_+*m*_7_
{**1**,**0**}	Survival	*m*_2_+(1−*m*_6_)*Bad*_tot_>*Bcl-x*_*L*_+*m*_7_
{**0**,**1**}	Survival	*m*_1_+*Bad*_tot_>*Bcl-x*_*L*_+*m*_7_
{**1**,**1**}	Apoptosis	*m*_2_+*Bad*_tot_>*Bcl-x*_*L*_+*m*_7_

Input in the format {*i,j*} denotes *p*53_killer_ =*i* × 2 × 10^5^,*Akt*_u_ =*j* × 2 × 10^5^. It is assumed that*m*_3_ ≪*m*_4_.

### Level of Bad (Bcl-x_L_) controls the transition between the logic gate AND and OR (AND* and OR)

**Figure 7:**
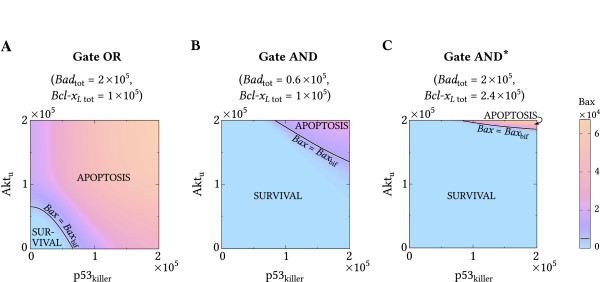
***Bax* in the input signals (*p53***_**killer**_**, *Akt***_**u**_**)-plane for three considered logic gates. (A)** gate OR, **(B)** gate AND, **(C)** gate AND*. For all gates the black curve is the apoptotic isoline *Bax = Bax*_bif_* = 5000*. For values of *Akt*_u_ and *p53*_killer_ above the *Bax = Bax*_bif_ isoline cell undergoes apoptosis, for values of *Akt*_u_ and *p53*_killer_ below this curve cell survives.

In Figure 7 we investigate system responses to*p53*_killer_ and*Akt*_u_ both in the range *(0, 2 × 10*^5^), assuming levels of*Bad*_tot_ and*Bcl-x*_*L* tot_ adequate to the gate type (Table 2). The*Bax = Bax*_bif_*=5000* isoline (white line) separates inputs {*p53*_killer_,*Akt*_u_} leading either to apoptosis or to survival. In gate OR (Figure 7A), apoptosis is activated for a relatively weak stimulation. It is triggered when steady state inputs satisfy*p53*_killer_*>0.66 × 10*^*5*^ or*Akt*_u_*>0.65 × 10*^*5*^, but even weaker signals, when in cooperation, may result in apoptosis. In contrast, in gate AND (Figure 7B) apoptosis requires cooperation of two stronger signals; for either*p53*_killer_*<0.84 × 10*^*5*^ or*Akt*_u_*<1.37 × 10*^*5*^ apoptosis cannot be initiated. In gate AND*, a higher*Akt*_u_ than in gate AND is required to trigger apoptosis (Figure 7C).

**Figure 8:**
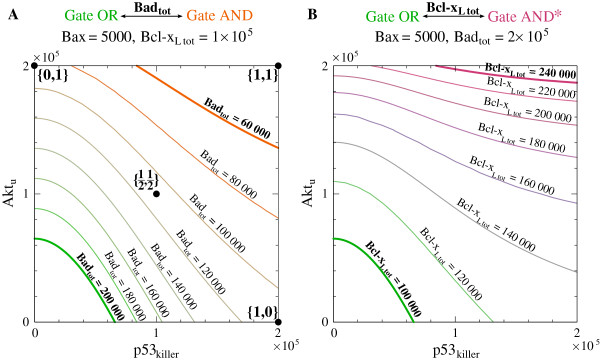
**Transitions between gate types. (A)** transition from gate OR to gate AND, **(B)** transition from gate OR to AND*. Apoptotic (*Bax = Bax*_bif_* = 5000*) isolines are plotted in the input signals (*p53*_killer_, *Akt*_u_)-plane. Black dots in **(A)** denote {*p53*_killer_, *Akt*_u_} pairs of input signals analyzed in Figures 9 and 10.

In Figure 8A we determined*Bax = 5000* isolines in the (*p53*_killer_,*Akt*_u_)-plane for different levels of*Bad*_tot_. For {*p53*_killer_,*Akt*_u_} “above” each isoline value the cell undergoes apoptosis, whenever its*Bad*_tot_ is greater or equal*Bad*_tot_ value for that isoline. Gate AND arises for*Bad*_tot_*≲0.9 × 10*^*5*^, while gate OR arises for$\text{Ba}d_{\text{tot}}≳1.1\times 10^{5}$. For*Bad*_tot_*∈(0.9 × 10*^*5*^,*1.1 × 10*^*5*^) the full dephosphorylation of Akt (*Akt*_u_*= Akt*_tot_) leads to apoptosis (for an arbitrary*p53*_killer_) but the increase of*p53*_killer_ to its highest assumed value ($p53_{\text{killer}}^{\text{max}}$) does not suffice for apoptosis without the additional signal from Akt.

The transition from OR to AND* gate can be analyzed analogously by varying*Bcl-x*_*L* tot_ (Figure 8B, Table 2).

### Apoptosis and survival in response to transient stimulation

In this section we determine the minimum duration of the stimulation phase (i.e. the phase in which one or two input signals {*p53*_killer_,*Akt*_u_} are present) needed to trigger apoptosis. We assume that before and after the stimulation phase*p53*_killer_*=0* and*Akt*_u_*=0*. The transient stimulation by p53_killer_ and/or Akt_u_ may cause that the caspase level passes a threshold above which the apoptotic decision is irreversible. This threshold caspase level (*≈2 × 10*^3^) is determined by the value of the unstable steady state for*Bax = 0*, see the bifurcation diagram (Figure 3).

**Figure 9:**
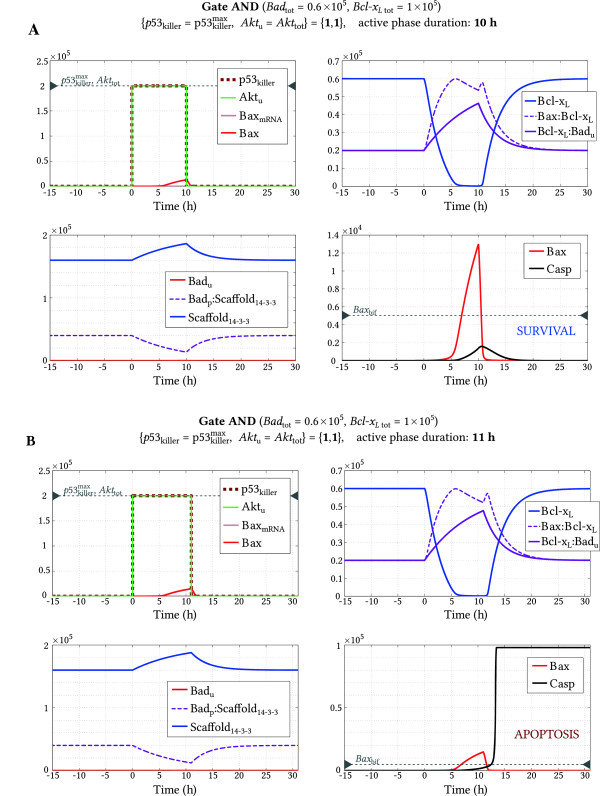
**Gate AND (*Bad***_**tot **_***= 0.6 × 10***^***5***^**, *Bcl-x***_***L* tot **_***= 1 × 10***^***5***^**) – transient apoptotic stimulation.** Input signal values correspond to point {**1**,**1**} in Figure 8. **(A)** 10 hours long stimulation is insufficient to trigger apoptosis; **(B)** 11 hours long stimulation suffices for triggering apoptosis.

For the gate AND we consider stimulation during which*Akt*_u_*= Akt*_tot_ and$p53_{\text{killer}}=p53_{\text{killer}}^{\text{max}}$. We know from the analysis presented in Figure 8A (point {**1**,**1**}) that such stimulation lasting sufficiently long leads to apoptosis. Here we estimated that the minimal duration of the stimulation phase is 10.5 hours. In Figure 9A we show that 10 hour long stimulation is insufficient for triggering apoptosis; despite*Bax* reaches *1.3 × 10*^*4*^*>Bax*_bif_ and *Casp* reaches *1.6 × 10*^3^*>Casp*_bif_, the system returns to the initial steady state of the low caspase level. In Figure 9B we show that after 11 hour long stimulation the caspase level passes the unstable steady state leading to the irreversible transition after which the caspases level settles at the high stable steady state. It is worth noting that after the stimulation*Bax* returns to its initial steady state. In the case of gate AND*, the minimal duration of the stimulation phase (for the same stimulation) is 20.5 hours.

**Figure 10:**
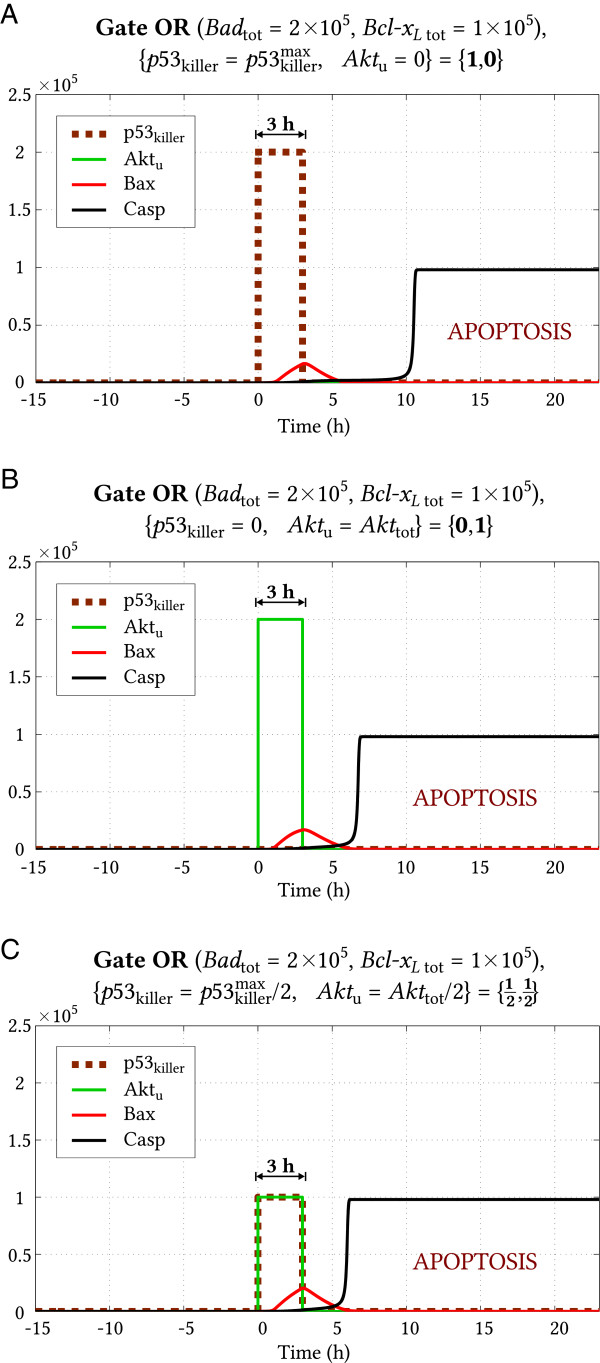
**Gate OR (*Bad***_**tot **_***= 2 × 10***^***5***^**, *Bcl-x***_***L* tot **_***= 1 × 10***^***5***^**) – apoptosis following 3 hours long stimulation.** Panels **(A)**, **(B)** and **(C)** correspond to input signal values shown in Figure 8 respectively by points {**1**,**0**}, {**0**,**1**} and {$\frac{1}{2}$,$\frac{1}{2}$}.

For gate OR we consider three particular modes of stimulation (Figure 10). In the first case we estimated that the minimum duration of the stimulation phase for the sole*p53*_killer_ signal ($\{p53_{\text{killer}}=p53_{\text{killer}}^{\text{max}}$,*Akt*_u_=0}, point {**1**,**0**} in Figure 8A) is 3.0 hours. In the case of Akt-only signaling ({*p53*_killer_=0,*Akt*_u_*=Akt*_tot_}, point {**0**,**1**} in Figure 8A) the critical duration of the stimulation phase is 2.8 hours. A slightly shorter stimulation (approximately 2.7 hours) is required when both signals are present simultaneously, even when their amplitudes are twice smaller than in the previous case ($\{p53_{\text{killer}}=\frac{1}{2}p53_{\text{killer}}^{\text{max}}$**,**$\text{Ak}t_{u}=\frac{1}{2}\text{Ak}t_{\text{tot}}\}$, point {$\frac{1}{2}$,$\frac{1}{2}$} in Figure 8A). In Figure 10 we show trajectories corresponding to the transition to apoptosis following 3 hours long stimulation in each of three cases discussed above.

## Discussion

Robust response to genotoxic stress is crucial for cell development and enables cells to preserve their genome integrity and suppress oncogenic transformation [2]. We proposed the ODE model of the apoptotic module, allowing for unambiguous cell death or survival decisions. The model was constructed on the basis of our current understanding of interactions of Bcl-2 family members in regulation of cell fate in response to stimuli mediated by two important upstream pathways of p53 and Akt. In the wider context, apoptosis is initiated in response to various stress stimuli, which often crosstalk with anti-apoptotic signals [90]; it is thus important to recognize, also from the mathematical point of view, how these signals might be integrated into survive-or-die decisions.

The Boolean modeling, which typically utilizes the qualitative knowledge about regulatory pathways, is often considered as a preliminary step to continuous models which require more detailed (kinetic) data, but allows also for a more precise description. Wittman and colleagues [91] introduced a systematic way of transforming Boolean models into ODE models by employing multivariate polynomial interpolation. Here, we show the reverse correspondence, demonstrating that the ODE model may behave akin to logic gates, in which YES or NO responses are associated with the change of relative abundances of proteins from pro- and anti-apoptotic subgroups of the Bcl-2 family, allowing for the release of the more abundant protein. Interestingly, we found that our logic gates are reconfigurable: levels of pro-survival and pro-apoptotic proteins (Table 2) together with seven macro-parameters (assumed constant in the model; Table 3) determine gate type, AND or OR (by relations given in Tables 4 and 5). Another example of reconfigurability was recently demonstrated by Goñi-Moreno and Amos [92].

The considered mitochondrial apoptotic module integrates pro-apoptotic signals from p53_killer_ and pro-survival signals from Akt. The final output of the module is the level of caspase-3, which (in the model) is activated by Bax (or Bak), but also auto-catalytically, which renders the apoptotic decision irreversible. After the caspase level surpassed unstable steady state, the presence of Bax is no longer needed, since caspase autoactivation suffices for their further build-up and execution of apoptosis. Caspase activation requires that free Bax surpasses a threshold of about 5000 molecules per cell, and remains over this level for sufficiently long time. The regulation of Bcl-2 family module can be summarized as follows: in resting cells, Bax (chosen as a representative for pro-apoptotic multidomain effectors) remains in the inactive form bound to Bcl-x_L_ (chosen as a representative for anti-apoptotic restrainers), while most of Bad (chosen as a representative for BH3-only pro-apoptotic proteins) is in the phosphorylated form bound to Scaffold_14-3-3_. The signal coming from p53 leads to the accumulation of Bax, while the signal coming from Akt (Akt dephosphorylation) results in Bad dephosphorylation. Dephosphorylated Bad may release Bax from Bcl-x_L_. In this way, both mechanisms contribute to the appearance of free Bax.

Cells characterized by high Bad level or/and low level of Bcl-x_L_, have relatively small reservoir of free Bcl-x_L_ (required to inhibit/sequester Bax) and thus are more prone to apoptosis. In these cells apoptosis follows from p53_killer_ accumulation or Akt dephosphorylation (gate OR); a BH3-domain mimetic, ABT-737, which mirrors binding capacities of Bad and engages pro-survival proteins (mostly Bcl-2), was shown to induce Bax/Bak-dependent killing [93]. Cells characterized by low Bad level or/and high level of Bcl-x_L_ have much larger reservoir of free Bcl-x_L_ and in these cells apoptosis requires both signals simultaneously (gate AND); this is consistent with experimental results showing that the overexpression of Bcl-x_L_ blocks apoptosis [94]. We demonstrated that transition between AND and OR gates results from either increase of Bad level or decrease of Bcl-x_L_ level, which confirms that levels of these proteins are important in regulating cell sensitivity to apoptosis.

It is known that Bad modifications, which interfere with Bad phosphorylation, can make cells more or less sensitive to apoptosis. Bad phosphorylation is limited by PRMT1-mediated methylation of two Bad arginine residues (Arg94 and Arg96), which prevents Akt-mediated phosphorylation of Bad at Ser99. Respectively, decreased methylation of Bad increases the fraction of phosphorylated Bad leading to its enhanced sequestration to Scaffold_14-3-3_, decreased caspase activity, and consequently enhanced cell viability [95,96].

In the therapeutic context, our findings suggest that in some (cancerous) cell lines or cell mutants (characterized by a low Bad level or a high Bcl-x_L_ level) apoptosis can result only from the simultaneous presence of both pro-apoptotic signals, i.e. elevated p53_killer_ level and Akt dephosphorylation (growth factor withdrawal). Radiotherapy against these cells which leads to the increase of p53_killer_ level (via DNA damage) should be accompanied by the inhibition of pro-survival Akt pathway in order to be effective. In turn, it suggests that cells characterized by high Bad level, or low Bcl-x_L_ level are very prone to apoptosis, which can follow even from growth factor withdrawal.

One could expect that levels or activities of proteins represented by Bad and Bcl-x_L_, which are assumed constant in the model, are also regulated in response to pro-apoptotic or pro-survival cues. Inclusion of these effects would expand the regulatory network and allow for the integration of a larger number of signals [86]. In order to improve the resolution of the presented apoptotic model, one should take into account individual characters of proteins from the group of effectors, restrainers and, most importantly, BH3-only proteins, and in this way cover additional effects such as the induction of expression of some BH3-only proteins by p53 [97,98], diverse specificities of BH3-only proteins with respect to various restrainers, and plausible requirement of the direct activation of effectors by some BH3-only proteins, which allows effectors to nucleate, oligomerize, and then release cytochrome *c*.

## Conclusion

We demonstrated that the mitochondrial apoptotic module may process signals in the way similar to logic gates OR and AND, which suggests a mechanism of the integration of apoptotic and survival signals before the death-or-survival decision is reached. The correspondence between ODEs and Boolean logic arises due to the high affinity, competitive binding of two subfamilies of pro-apoptotic proteins, multidomain effectors (represented by Bax) and BH3-only proteins (represented by Bad) to anti-apoptotic restrainers (represented by Bcl-x_L_). Such regulation is based on stoichiometry of interacting proteins, and introduces dynamical thresholds for all protein levels, which, when surpassed, change output from NO to YES. Specifically, apoptosis arises when the level of anti-apoptotic restrainers is surpassed by the combined levels of pro-apoptotic unphosphorylated BH3-only proteins (controlled by Akt) and pro-apoptotic effectors (controlled by p53). We demonstrated that the transition between OR and AND gates is accomplished by the change of a level of single component (either decrease of pro-apoptotic protein Bad or increase of pro-survival Bcl-x_L_), without any modification of topology of the network or even kinetic rate parameters.

## Competing interests

The authors declare that they have no conflict of interest.

## Authors’ contributions

TL and MK conceived of the study. MNB, BH and MK analyzed the model and performed simulations. All authors wrote and approved the manuscript.

## Supplementary Material

Additional file 1 Matlab source code. Click here for file
